# Primo Vascular System in the Subarachnoid Space of a Mouse Brain

**DOI:** 10.1155/2013/280418

**Published:** 2013-05-28

**Authors:** Sang-Ho Moon, Richard Cha, Geo-Lyong Lee, Jae-Kwan Lim, Kwang-Sup Soh

**Affiliations:** ^1^Nano Primo Research Center, Advanced Institute of Convergence Technology, Seoul National University, Suwon 443-270, Republic of Korea; ^2^Department of Research & Development, Peace World Medical Co., Ltd., Seoul 110-775, Republic of Korea; ^3^Nadi Primo Research Institute, Graduate School of Integrative Medicine, Sun Moon University, Asan-si 336-708, Republic of Korea; ^4^College of Physical Education, The University of Suwon, Hwaseong-si, Gyeonggi-do 445-743, Republic of Korea; ^5^Korea Institute of Oriental Medicine, Daejeon 305-811, Republic of Korea

## Abstract

*Objective*. Recently, a novel circulatory system, the primo vascular system (PVS), was found in the brain ventricles and in the central canal of the spinal cord of a rat. The aim of the current work is to detect the PVS along the transverse sinuses between the cerebrum and the cerebellum of a mouse brain. *Materials and Methods*. The PVS in the subarachnoid space was analyzed after staining with 4′,6-diamidino-2-phenylindole (DAPI) and phalloidin in order to identify the PVS. With confocal microscopy and polarization microscopy, the primo vessel underneath the sagittal sinus was examined. The primo nodes under the transversal sinuses were observed after peeling off the dura and pia maters of the brain. *Results*. The primo vessel underneath the superior sagittal sinus was observed and showed linear optical polarization, similarly to the rabbit and the rat cases. The primo nodes were observed under the left and the right transverse sinuses at distances of 3,763 **μ**m and 5,967 **μ**m. The average size was 155 **μ**m × 248 **μ**m. *Conclusion*. The observation of primo vessels was consistent with previous observations in rabbits and rats, and primo nodes under the transverse sinuses were observed for the first time in this work.

## 1. Introduction

The primo vascular system (PVS) was proposed by Kim as a third circulatory system that corresponded to and extended the acupuncture meridians [1]. In various parts of an animal's body, the PVS was confirmed [2], especially in blood vessels [3], the heart [4], lymph vessels [5, 6], and on the surfaces of internal organs [7] of mice, rats, and rabbits. 

The PVS in the central nervous system was first observed in the 4th ventricle of the brain and the central canal of the spinal cord of a rabbit by using a staining dye, chrome hematoxylin [8], and subsequently in the 3rd ventricle of a rat brain by using Trypan blue dye [9]. Fluorescent nanoparticles were injected into the lateral ventricle of a rat to detect the PV in the fourth ventricle and the spinal cord of a rat [10]. The PVS in the subarachnoid space of a rat spine [11] and in the sciatic nerve of a rat [12] has also been reported. The function of the PVS with respect to nerve regeneration and acupuncture is not yet studied [13, 14].

Recently, the PVS was observed on the pia mater of a rat brain by using Alcian blue [15]. Primo vessels (PVs) and primo nodes (PNs) were found underneath the superior sagittal sinus (SSS) in the sagittal fissure of a rabbit [16]. The PV underneath the SSS was also found in a rat, and it showed a strong linear optical polarization.

Thus, an investigation as to whether the PV in the falx cerebri underneath the SSS also exists in mice and exhibits a similar polarization effect is needed. Another pending effort is to find PNs in a mouse brain. The present work reports observations of a PV underneath the SSS and its optical polarization and observations of PNs on the transverse sinuses instead of the SSS.

## 2. Materials and Methods

### 2.1. Animal Preparation

Eleven adult ICR mice (female, 10 weeks old, 33 g) were purchased from Dooyeol Biotech Co., Ltd., (Seoul, Korea). Animals were housed in the laboratory animal facility at 25°C and 60% relative humidity under a 12-hour light/dark cycle. Procedures involving the animals and their care were in full compliance with current international laws and policies (Guide for the Care and Use of Laboratory Animals, National Academy Press, 1996). All surgical procedures were performed under general anesthesia (25-mg/kg Zoletil and 10-mg/kg Rompun administered by intramuscular injection).

### 2.2. Brain Specimen Preparation

After the mouse had been sacrificed by overanesthetizing, the mouse was decapitated, and the head was fixed by putting it in paraformaldehyde (PFA, Sigma-Aldrich, USA) for one week in the refrigerator (2°C). The head was opened one hour before the experiment and was washed with running tap water. After the skull had been separated from the brain by using tweezers, we carefully kept the dura mater and the pia mater of the brain intact. Before peeling the dura mater off the brain, as a preparation for detection of primo nodes and primo vessels with the features of nuclei distribution, we sprayed 4′,6-diamidino-2-phenylindole (DAPI) solution on the surface of the dura mater and the pia mater of the brain. Because the dura mater had a high density of nuclei, discerning the features of the nuclei distribution in the PVS that was covered by the dura mater was difficult. Therefore, we developed techniques to peel the dura mater off the brain meninges. In this method, we were able to discern the PNs, PVs on the pia mater of the brain.

### 2.3. Staining and Observations with Microscopes

We applied DAPI and phalloidin reagents for staining of nuclei and f-actin molecules in the cells, respectively. After a one-hour DAPI (Invitrogen, ProLong Gold Antifade Reagent with DAPI, USA) staining, we washed the sample with phosphate based saline (PBS) three times. After that, the phalloidin (Invitrogen, Rhodamine Phalloidin, USA) staining was done for one hour, and the PBS washing was done three times.

After the samples had been washed, we dried them and poured on Neomount (M1289-10 mL, Sigma-Aldrich, USA) solution on the brain with care not to make bubbles and noise so that the Neomount would not become too thick for analysis of the primo vessels with a high-magnification microscope. We gently placed the cover glass on the sample; the cover should be maintained level for high-magnification observation.

The stained specimens were investigated with a phase-contrast microscope (BX51, Olympus, Japan) and polarization microscope (KSM-BA3, Samwon, Korea) to search for loose connective tissues, such as blood capillaries, nerve tissues, and bundles of PVs and PNs. A fluorescent microscope (MVX10, Olympus, Japan) was used to investigate the characteristic features of the nuclei and the F-actin distributions of the PVs and the PNs with DAPI and phalloidin staining, respectively. Confocal laser scanning microscopy (C1 plus, Nikon, Japan) was used to optically scan the threadlike PVs to uncover the characteristic bundle structure of the PVs.

## 3. Results

**Figure 1:**
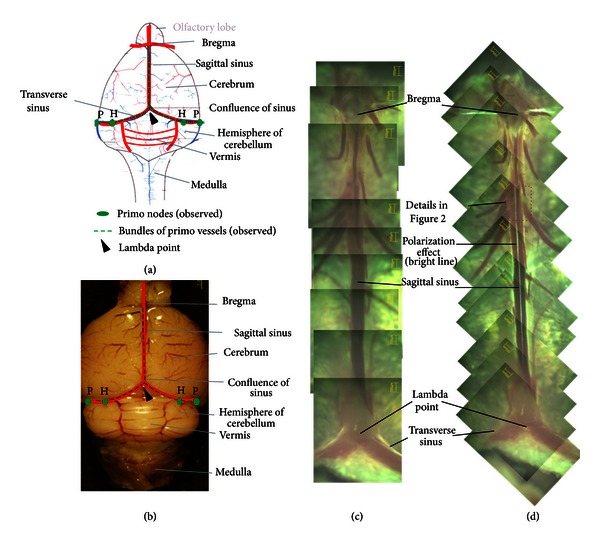
(a) Schematic illustration of the cranial view of a mouse brain. The sagittal sinus branches to two transverse sinuses at the confluence of sinuses where the *λ* point (arrow head) lies. The primo vessels ran along the sagittal sinus and branches and then along the two transverse sinuses (dotted line). The primo nodes were observed at the points designated as H and P on two sides of the brain. H is at the point where the hemisphere of the cerebellum and the vermis meet along the transverse sinus. P is further outside, along the transverse sinus near the end of the hemisphere of the cerebellum. The distances from the *λ* point to the H-point and the P-point were 3,763 *μ*m and 5,967 *μ*m, respectively. (b) Stereo microscopic image of the cranial view of a mouse brain. The symbols are the same as in (a). (c) Polarization microscope image of brain meninges complex of the dura and the pia mater peeled off from the brain and put on a slide with the pia mater side down. (d) Polarization microscope image of the same specimen rotated 45 degrees relative to (c). There appeared a strong polarization signal along the midline of the sagittal sinus. The polarization image was in agreement with those in the rabbit and the rat brains. The image shows a primo vessel in the falx cerebri under the dura mater of the superior sagittal sinus.

**Figure 2:**
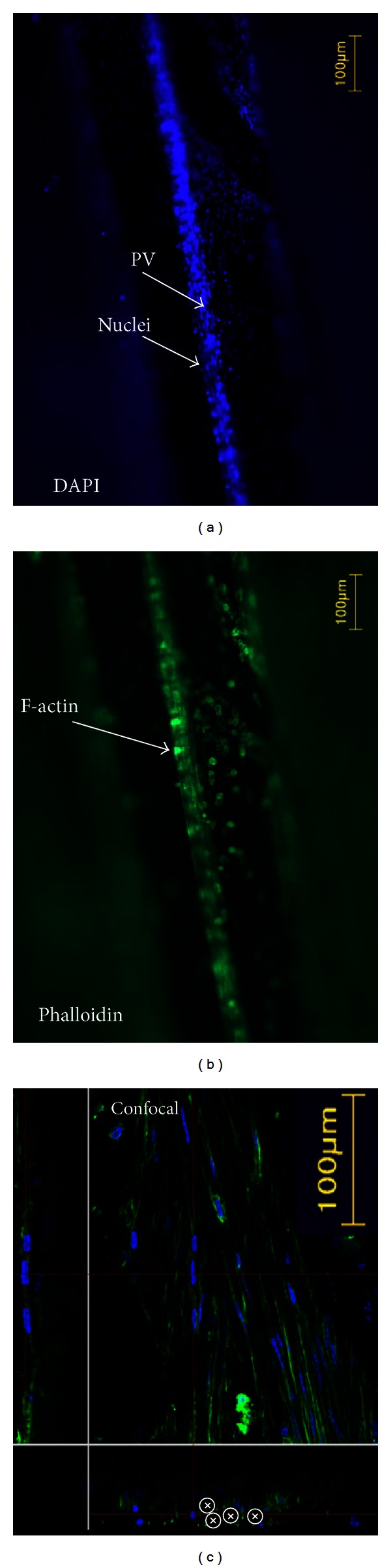
(a) Nuclei distribution in the primo vessel in the falx cerebri stained with DAPI. The dark neighboring background is the sagittal sinus. The specimen is the dotted box area of Figure 1(d). (b) F-actin distribution in the same specimen stained with phalloidin. (c) Confocal laser scanning microscope image of the same specimen. The left column shows a longitudinal section, which reveals rod-shaped nuclei. The bottom panel shows a transversal section of the primo vessel, which reveals multiple channels *ⓧ*. These two features are in agreement with data obtained from rabbit and rat brains.

A schematic illustration and a real specimen of the mouse brain are shown in Figures 1(a) and 1(b), respectively. The falx cerebri houses the superior sagittal sinus. The PV was located in the falx cerebri and could be detected because of its strong polarization signal, as shown in Figures 1(c) and 1(d). We confirmed the representative features of a PV by staining the sample with DAPI and phalloidin to reveal the nuclei and the f-actin distributions. The results are presented in Figures 2(a), 2(b), and 2(c). The confocal laser scanning microscope image shows the presence of rod-shaped nuclei and multiple bundle of channels, as shown with the asterisk *ⓧ* in the lower panel of Figure 2(c).

We observed PNs at two locations in the subarachnoid space between the pia mater and the arachnoid mater of a mouse brain: the first location, named as the H-point, is under the transverse sinus at the boundary between the hemisphere and the vermis of the cerebellum. The second location, named as the P-point, is further outside along the transverse sinus near the end of the hemisphere of the cerebellum. The average distances from the H-point and the P-point to the *λ* (lambda) point were 3,763 *μ*m and 5,967 *μ*m (Figure 1(a)).

As shown in Table 1, we did experiments with eleven mice and found six PNs at the left H-point, six at the right H-point, three at the left P-point, and three at the right P-point. The average distances from the *λ* point and the average size were as follows:

 3,925 *μ*m (distance); 125 *μ*m (short axis) × 230 *μ*m (long axis), the left H-point,
 3,600 *μ*m; 120 *μ*m (short axis) × 205 *μ*m (long axis), the right H-point,
 5,533 *μ*m; 177 *μ*m (short axis) × 350 *μ*m (long axis), the left P-point, and
 6,400 *μ*m; 197 *μ*m (short axis) × 207 *μ*m (long axis) the right H-point.

**Figure 3:**
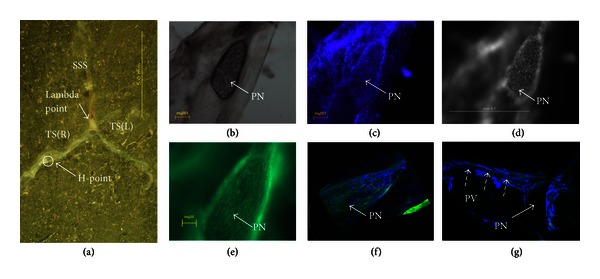
(a) Stereomicroscopic image of the dura mater taken from the mouse brain. The PN is indicated with a circle that is 3,900 *μ*m from the *λ* point (arrow). The specimen is sample no. 10 in Table 1. PV: primo vessel, PN: primo node, SSS: superior sagittal sinus, and TS (R, L): transverse sinus right, left, respectively. (b) Phase-contrast microscopic image of the PN. It has an eggplant shape, and its size is 180 *μ*m × 420 *μ*m. (c) Fluorescent image of the nuclei distribution of the same PN after DAPI staining. The DAPI staining was not clearly seen with the phase-contrast microscope because the PN was too thick. (d) The fluorescence microscope with tissues MVX-10 showed a better image of the DAPI staining. (e) The f-actins of cells in the same PN are shown with phalloidin staining. (f) A 3D image of the PN stained with DAPI and phalloidin taken with a confocal laser scanning microscope. (g) The primo vessel (PV) attached to the PN is shown. Primo vessel (dotted arrow).

**Figure 4:**
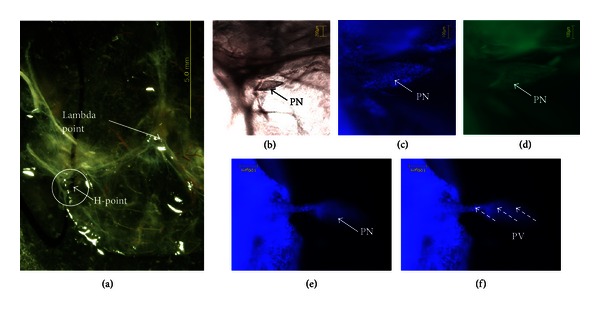
(a) Another specimen of the PN located in the circle shown in the stereomicroscopic image of the dura mater and the pia mater of a mouse brain (sample no. 11 in Table 1). It was located 4,800 *μ*m from the *λ* point. (b) Phase-contrast microscope images of the PN. It had a fusiform shape, and its size was 200 *μ*m × 400 *μ*m. (c) and (d) Fluorescence images of the PN with DAPI and phalloidin staining, respectively. (e) and (f) Fluorescence images of the PV attached to the PN with DAPI and phalloidin staining, respectively. The pia mater that covered the PV was removed to reveal the PV. Rod-shaped nuclei and f-actin distributions were noticed.

**Figure 5:**
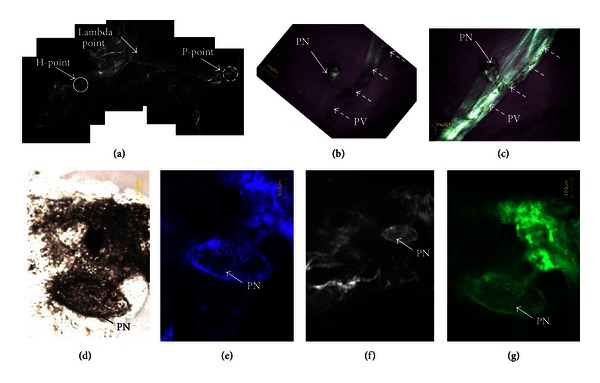
(a) Stereo microscopic image of a dura mater with two PNs indicated with solid and dotted circles, which were 3,600 *μ*m and 5,400 *μ*m from the *λ* point (arrow), respectively. (b) Polarization microscopic images at a 45-degree rotation. The image in (c) has bright polarization signals along the transverse sinus, as seen in Figure 1(d). (d)–(g) Phase-contrast, DAPI-stained, MVX-10, and phalloidin-stained PN images, respectively, of the PN in the dotted circle. Its shape was oval and its size was 230 *μ*m × 460 *μ*m.

Remarkably PNs of fairly distinguishable shapes (Figures 3, 4, and 5) were repeatedly observed at the H-points and the P-points along the transverse sinuses.

A detailed description of a PN at the H-point is shown in Figure 3. The location in the dura mater was determined under a stereomicroscope (Figure 3(a)), and the PN specimen was observed with a phase-contrast microscope equipped for fluorescence. The observation after DAPI and phalloidin staining revealed cells and nuclei packed in the PN, as expected (Figures 3(b), 3(c), 3(d), 3(e)). A more realistic view was obtained with a confocal laser scanning microscope as presented in Figure 3(f). The PV attached to the PN showed rod-shaped nuclei aligned along the PV (Figure 3(g)).

Another case of a PN at the H-point is presented to show that its apparent shape is different from the previous shape (Figures 4(b), 4(c), and 4(d)). In this example, we exposed the PN and its attached PV by removing the surrounding dura and pia maters (Figures 4(e) and 4(f)). The DAPI signals showed packed nuclei in the PN and rod-shaped nuclei in the PV.

A PN at the P-point was observed, as indicated with the dotted circle in Figure 5(a). Its shape was oval, and its size was 230 *μ*m × 460 *μ*m. Its phase-contrast, DAPI-stained, MVX-10, and phalloidin-stained images (Figures 5(d), 5(e), 5(f), and 5(g), resp.) were similar to those for the PNs at the H-points. A PN at the H-point is indicated with a circle in Figure 5(a). Figures 5(b) and 5(c) demonstrate a strong polarization signal along the PV attached to the PN.

## 4. Discussion

The PV in the falx cerebri embedded in the dura mater under the sagittal sinus was first observed in a rabbit brain [15] and subsequently in a rat brain. The current work confirmed the presence of a similar PV in a mouse brain. The thicknesses of the PVs were 55.7 *μ*m, 56.7 *μ*m, and 31 *μ*m in the cases of rabbits, rats, and mice, respectively. The distributions of rod-shaped nuclei and the bundle structures of the multiple channels were similar.

The linear polarization of the PV that was previously observed in a rat brain was also clearly visible in the mouse case. This optical property was useful in detecting the PNs along the transverse sinus, as shown in Figure 5.

The presence of a PN in the brain was noticed in the case of a rabbit at the confluence of the transverse sinuses, that is, near the *λ* point [15]. However, no PN was detected at a similar location in mice. In our experiment, PNs were observed at completely different positions, the left and the right H-points and the left and the right P-points along the transverse sinuses. The sizes of the PNs were 286 *μ*m × 503 *μ*m and 155 *μ*m × 248 *μ*m in rabbits and mice, respectively. The PNs of both animals were packed with many cells inside.

PNs packed with various cells were a common feature. Especially, immune cells were abundant in the PNs on the surfaces of internal organs [17] and inside lymph vessels [18]. Immunohistochemical data also suggested the presence of embryonic-like stem cells in the PNs on the surfaces of internal organs and inside the lymph vessels [18]. Furthermore, observations of cancer stem cells in the PNs of xenografted cancer mice were recently reported [19].

In the current work, we were not able to isolate and analyze the cells from the brain PNs. It remains an important question whether the PNs in brain contain abundant immune cells and/or stem cells as observed in the PNs on the surfaces of internal organs. There are also critical works against the acupuncture relevance of the PVS [20]. These medically critical features are worthy of more future work.

## Figures and Tables

**Table 1:** Primo nodes in the mouse brain observed in this experiment.

Subject		Locations and Primo nodes		Sizes
No.	Weight (g)	Location	Distance from *λ* point (*μ*m)	Short (*μ*m) × long (*μ*m)
1	32	Left H-point	3,600	120 × 190
2	34	Left H-point	4,000	170 × 300
		Right H-point	3,500	200 × 270
3	35	Right H-point	3,500	50 × 80
4	32	Left H-point	3,700	30 × 110
		Right H-point	3,300	50 × 80
5	31	Right P-point	7,800	250 × 400
6	32	Left H-point	3,750	130 × 220
7	35	Left H-point	3,700	100 × 160
		Left P-point	5,500	80 × 190
8	32	Right H-point	3,800	120 × 180
		Right P-point	5,900	190 × 230
9	34	Right H-point	3,600	120 × 200
		Left P-point	5,400	230 × 460
10	31	Right H-point	3,900	180 × 420
		Left P-point	5,700	220 × 400
		Right P-point	5,500	150 × 180
11	31	Left H-point	4,800	200 × 400
Average	33	Left H-point	3,925	125 × 230
		Right H-point	3,600	120 × 205
		Left P-point	5,533	177 × 350
		Right P-point	6,400	197 × 207

The subjects were 10-week-old female ICR mice. The primo nodes had cucumber or oval shapes; short and long axes were measured.

## References

[1] Kim BH (1965). The Kyungrak system. *Journal of Jo Sun Medicine*.

[2] Avijgan M (2013). Can the primo vascular system (Bong Han Duct System) be a basic concept for qi production?. *International Journal of Integrative Medicine*.

[3] Johng HM (2005). *Observation method for threadlike structures inside blood and lymph vessels and on organ surfaces. Study on extra-curriculum teaching model of biophysics [Ph.D. thesis]*.

[4] Lee BC, Kim HB, Sung B (2011). Network of endocardial vessels. *Cardiology*.

[5] Noh YI, Rho M, Yoo YM, Jung S, Lee SS (2012). Isolation and morphological features of primo vessels in rabbit lymph vessel. *Journal of Acupuncture and Meridian Studies*.

[6] Choi I, Chung HK, Hong YK, Soh KS, Kang KA, Harrison D (2011). Detection of the primo vessels in the rodent thoracic lymphatic ducts. *The Primo Vascular System: Its Role in Cancer and Regeneration*.

[7] Lee KJ, Kim S, Jung TE, Jin D, Kim DH, Kim HW (2004). Unique duct system and the corpuscle-like structures found on the surface of the liver. *Journal of International Society of Life Information Science*.

[8] Lee BC, Kim S, Soh KS (2008). Novel anatomic structures in the brain and spinal cord of rabbit that may belong to the Bonghan system of potential acupuncture meridians. *Journal of Acupuncture and Meridian Studies*.

[9] Dai JX, Lee BC, An P (2011). *In situ* staining of the primo vascular system in the ventricles and subarachnoid space of the brain by trypan blue injection into the lateral ventricle. *Neural Regenerational Research*.

[10] Lim JK (2011). *Visualization of primo vascular system in brain and spinal cord with fluorescent nanoparticles [Ph.D. thesis]*.

[11] Lee IH, Su Z, Kim KW, Lee BC, Soh KS, Soh KS, Kang KA, Harrison D (2011). Visualization of the primo vascular system by using trypan blue in the subarachnoid space of rats. *The Primo Vascular System: Its Role in Cancer and Regeneration*.

[12] Jia ZF, Lee BC, Eom KH (2010). Fluorescent nanoparticles for observing primo vascular system along sciatic nerve. *Journal of Acupuncture and Meridian Studies*.

[13] Chang IA, Namgung U (2013). Induction of regenerative responses of injured sciatic nerve by pharmacopuncture therapy in rats. *Journal of Acupuncture and Meridian Studies*.

[14] Park ES, Kim HY, Youn DH (2013). The primo vascular structures alongside nervous system: its discovery and functional limitation. *Evidence-Based Complementary and Alternative Medicine*.

[15] Lee HS, Lee BC (2012). Visualization of the network of primo vessels and primo nodes above the pia mater of the brain and spine of rats by using alcian blue. *Journal of Acupuncture and Meridian Studies*.

[16] Nam MH (2013). *Investigation of the primo vascular system underneath the superior sagittal sinus in the brain [M.S. thesis]*.

[17] Lee BC, Yoo JS, Ogay V (2007). Electron microscopic study of novel threadlike structures on the surfaces of mammalian organs. *Microscopy Research and Technique*.

[18] Kwon BS, Chang MH, Yu SS, Lee BC, Ro JY, Hwang S (2012). Microscopic nodes and ducts inside lymphatics and on the surfaces of internal organs are rich in granulocytes and secretory granules. *Cytokine*.

[19] Islam A, Thomas S, Sedoris K, Miller D (2012). Tumor-associated primo vascular system is derived from xenograft, not host. *Experimental and Molecular Pathology*.

[20] Wang XY, Shi H, Shang HY (2012). Are primo vessels(PVs) on the surface of gastrointestine involved in regulation of gastric motility induced by stimulation acupoints ST36 or CV 12?. *Evidence-Based Complementary and Alternative Medicine*.

